# A Novel Quantitative Fluorescent Reporter Assay for RAG Targets and RAG Activity

**DOI:** 10.3389/fimmu.2013.00110

**Published:** 2013-05-16

**Authors:** Inês Trancoso, Marie Bonnet, Rui Gardner, Jorge Carneiro, Vasco M. Barreto, Jocelyne Demengeot, Leonor M. Sarmento

**Affiliations:** ^1^Instituto Gulbenkian de CiênciaOeiras, Portugal

**Keywords:** recombination-activating gene 1, V(D)J recombination, green fluorescent proteins, reporter, inversion

## Abstract

Recombination-Activating Genes (RAG) 1 and 2 form the site specific recombinase that mediates V(D)J recombination, a process of DNA editing required for lymphocyte development and responsible for their diverse repertoire of antigen receptors. Mistargeted RAG activity associates with genome alteration and is responsible for various lymphoid tumors. Moreover several non-lymphoid tumors express RAG ectopically. A practical and powerful tool to perform quantitative assessment of RAG activity and to score putative RAG-Recognition signal sequences (RSS) is required in the fields of immunology, oncology, gene therapy, and development. Here we report the detailed characterization of a novel fluorescence-based reporter of RAG activity, named GFPi, a tool that allows measuring recombination efficiency (RE) by simple flow cytometry analysis. GFPi can be produced both as a plasmid for transient transfection experiments in cell lines or as a retrovirus for stable integration in the genome, thus supporting *ex vivo* and *in vivo* studies. The GFPi assay faithfully quantified endogenous and ectopic RAG activity as tested in genetically modified fibroblasts, tumor derived cell lines, developing pre-B cells, and hematopoietic cells. The GFPi assay also successfully ranked the RE of various RSS pairs, including *bona fide* RSS associated with V(D)J segments, artificial consensus sequences modified or not at specific nucleotides known to affect their efficiencies, or cryptic RSS involved in RAG-dependent activation of oncogenes. Our work validates the GFPi reporter as a practical quantitative tool for the study of RAG activity and RSS efficiencies. It should turn useful for the study of RAG-mediated V(D)J and aberrant rearrangements, lineage commitment, and vertebrate evolution.

## Introduction

V(D)J recombination, the somatic rearrangement of variable (V), diversity (D), and joining (J) segments of the antigen receptor genes, is the phenomenon responsible for the very large diversity of the B and T cell antigen receptors in jawed vertebrates (Tonegawa, 1983). The Recombination-Activating Genes (RAG) 1 and 2 form the endonuclease that specifically recognizes recombination signal sequences (RSSs) adjacent to each gene segment and generates DNA double strand breaks (DSBs) (Schatz et al., 1989; Oettinger et al., 1990). In a typical V(D)J reaction, the non-homologous end-joining (NHEJ) machinery subsequently processes these DSBs to produce a functional gene. On one side the signal ends retain the RSSs perfectly joined together, whereas the other breaks, named coding ends, are edited prior to ligation, thus creating the junctional diversity that is the signature of V(D)J recombination (Bassing et al., 2002).

The RSS comprises a heptamer and a non-amer separated by a 12 or 23 nucleotide spacer sequence (Gellert, 2002). The heptamer and nonamer, but most notably, the spacer sequences are considerably degenerated, which favors a wide range of interactions with RAG and the fine-tuning of the rearrangement efficiency that is important for the generation of antigen receptor diversity (Cowell et al., 2004). However, a consequence of such degeneracy is that sequences similar to RSSs are found outside of antigen receptor loci. These are named cryptic RSSs (cRSS) and their targeting by RAG has been associated with tumorigenesis (Marculescu et al., 2006; Schlissel et al., 2006). Thus, V(D)J recombination challenges genome integrity and has to be kept under tight control at multiple regulatory levels.

As the breakthrough of *RAG1* cloning relied on a reporter plasmid for V(D)J (Schatz et al., 1989; Oettinger et al., 1990), it is not surprising that a number of additional reporters have been designed over the years. To study RAG1/2 activity in eukaryotic cells and to avoid the intricate multi-step of biochemical or prokaryote-based assays (Hesse et al., 1987), reporters in which the readout is based on gain of fluorescence became popular (Liang et al., 2002; Borghesi et al., 2004; Zheng and Schwarz, 2006; Arnal et al., 2010; Scott et al., 2010; Lutz et al., 2011). While each of these reporters has appealing features, none combines a double-fluorescence readout for the presence of the plasmid and its recombined form, the flexibility for transient or stable assays as an episomal or retroviral substrate, and high resolution in detecting RAG-mediated recombination events. In this work, we describe a novel retroviral-fluorescent reporter of RAG activity (GFPi) and test its function *in vitro* and *in vivo*, in stable and transient recombination assays. This tool is sensitive and practical as it allows rapid and quantitative measurement of RAG activity in assays sensitive to RSS nucleotide composition.

## Materials and Methods

### RAG expression constructs

**Figure 1:**
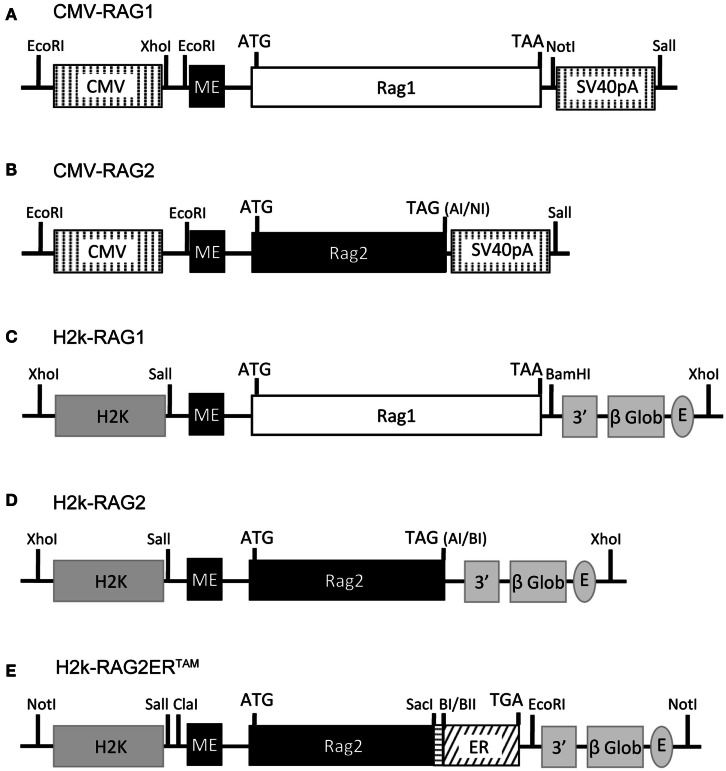
**RAG1/2 expression constructs**. Structure of the CMV-RAG1 **(A)**, CMV-RAG2 **(B)**, H2k-RAG1 **(C)**, H2k-RAG2 **(D)**, H2k-RAG2-ER^TAM^ **(E)** plasmids. RAG1 **(A,C)** and RAG2 **(B,D)** constructs contain genomic fragments spanning the whole RAG1 (white box) or RAG2 (black box) coding sequences (ATG and stop codons indicated). For the RAG2-ER^TAM^ construct **(E)**, the genomic fragment spanning the RAG2 coding sequence (long black box; ATG indicated) was truncated at the 3′ end, fused to a PCR fragment (horizontal dashed box) used to restore the RAG2 full length without the stop codon and then fused in frame with the coding sequence of the G525R mutant form of the estrogen receptor (ER, slant dashed box) that ends in the stop codon (TGA). The CMV expression cassette **(A,B)** contains the CMV viral promoter (5′ vertical dashed box), an engineered first exon of the *RAG2* gene (*RAG2* miniexon; ME, small black box) and the SV40-polyA (3′ vertical dashed box). The H2k expression cassette **(C,D)** contains the H2k mouse MHC class I promoter (5′ gray box), the *RAG2* miniexon (ME, small black box), the 3′ untranslated region of the human β-globin gene, including a splice site and the polyA (3′ gray boxes), and the Eμ enhancer (gray oval). Restriction sites are identified and depicted as vertical lines (except for: AI, *Ase*I; NI, *Not*I; BI, *Bam*HI; and BII, *Bgl*II).

The CMV-RAG, H2k-RAG, and the H2k-RAG2ER^TAM^ expression vectors are depicted in Figure 1. The CMV-RAG expression plasmids were generated in the pCMVβ vector (Clontech) by inserting an *Xho*I-*Not*I fragment containing the first exon of the murine *RAG2* gene (*RAG2* miniexon) adjacent to the *Xcm*I-*Xcm*I murine *RAG1* genomic sequence or a *Xho*I-*Ase*I fragment containing the *RAG2* miniexon adjacent to the *Sal*I-*Ase*I murine *RAG2* genomic sequence. In the latter case, the stop codon was regenerated upon blunt-end ligation. RAG gene segments are described in (Barreto et al., 2001) where construction of the H2k-RAG vectors is reported.

The H2k-RAG2ER^TAM^ plasmid was constructed from the DRII-6 plasmid (Oltz et al., 1993) and the pcDNA3-ER^C^ vector (kindly provided by P. Coffer). A first intermediate construction, pKS-RAG2 contained the *Pst*I-*Xho*I *RAG2* miniexon and the *RAG2* *Sal*I-*Not*I fragments inserted into pBluescript II KS(−) (Stratagene). Next, pKS-RAG2DStop, contained a *Sac*I digested PCR fragment of the 3′ region of the RAG2 coding sequence generated with the following primers: forward – 5′-TCAACGGAGCTCAATAAACC-3′ overlapping the *RAG2* *Sac*I restriction site (underlined); reverse – 5′-TGAGG*A*GCT*C*TTGCTAAAT*AG*ATC*T*AACAGTCTTCTAAGG-3′ with altered nucleotide positions (italic) creating the restriction sites (underlined) *Bgl*II, that disrupts the stop codon, and a second *Sac*I site at the 3′ end. The amplicon replaced the *Sac*I fragment of the pKS-RAG2. The next intermediate construct, pcDNA3-RAG2ER^TAM^ contained a *Sal*I-*Bgl*II fragment from pKS-RAG2DStop ligated into the *Hin*dIII-*Bam*HI digested pcDNA3-ER^C^ vector to generate the in-frame fusion of *RAG2* with the G525R mutant form of the hormone binding domain of the estrogen receptor. The final H2k-RAG2ER^TAM^ construct was obtained by inserting a *Cla*I-*Not*I fragment from pcDNA3-RAG2ER^TAM^ into a modified version of pHsE3′ digested with *Cla*I-*Eco*RV.

### GFPi constructions

The GFPi reporters of RAG-mediated inversion were built on the previously described pMigR1, a MSCV-internal ribosomal entry site (IRES)-GFP retroviral vector (Pear et al., 1996).

**Figure 2:**
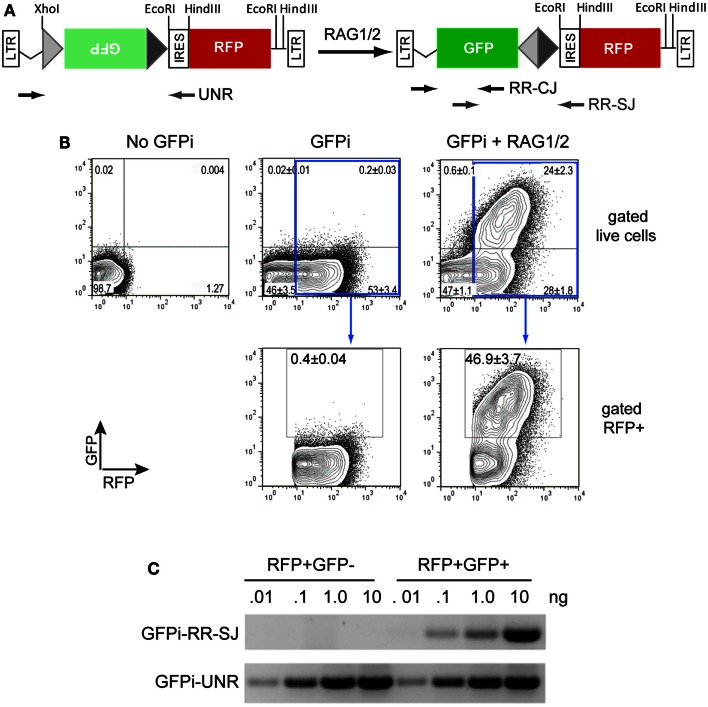
**The GFPi reporter**. **(A)** Linear representation of the GFPi reporter (left) and resulting structure following RAG activity (right); GFPi is constructed from a murine stem cell virus (MSCV) retroviral vector and the portion flanked by the long-terminal repeats (LTRs) is represented. From the 5′ to the 3′ LTR, the relevant segments are as follow: a 12-spacer RSS (light gray triangle), the GFP inverted complementary sequence (green box), a 23-spacer RSS (dark gray triangle), an internal ribosomal entry site (IRES), the RFP coding sequence (red box). Upon RAG-mediated GFP inversion formation of a coding joint (CJ) and signal joint (SJ) is indicated. The arrows represent the primers used to detect GFPi unrearranged (UNR) and rearranged (RR) CJ and SJ in **(C)**. **(B)** GFP/RFP flow cytometry contour plot analysis of a representative *in vitro* recombination assay. 293T cells were transfected with 10 μg DNA containing either only irrelevant DNA (no GFPi), 5 μg GFPi (GFPi) alone or together with 0.8 μg CMV-RAG1 and 0.7 μg CMV-RAG2 (GFPi + RAG). GFPi carried consensus 12 and 23-RSS. Upper plots are gated on live cells while lower plots are in addition gated on RFP^+^ cells. Values represent the percentage of each cell population in the quadrant (upper plots) or in the GFP^+^ gate (lower plots, *n* = 4). **(C)** Semi-quantitative PCR analysis of specific amplicons to detect the rearranged signal joint region of GFPi (GFPi-RR-SJ), or unrearranged GFPi (GFPi-UNR) performed with the indicated plasmid amounts isolated from sorted RFP^+^GFP^+^ or RFP^+^GFP^−^ 293T cells previously transfected with GFPi + RAG or the GFPi alone, respectively.

We first constructed the GFPi-red fluorescent protein (RFP) (Figure 2). In the intermediate construction, MSCV-IRES-RFP, the monomeric RFP (mRFP) from pRSETB-mRFP1 (Campbell et al., 2002) replaces GFP in pMigR. The backbone of pMigR1 released from the IRES-GFP fragment by *Eco*RI and *Hin*dIII digestion was ligated to a pMigR1 *Eco*RI–*Nco*I(blunt) fragment containing the IRES sequence together with a pRSETB-mRFP1 *Bam*HI(blunt)–*Hin*dIII fragment containing the mRFP sequences. The next cloning step consisted in inserting GFP bordered or not with RSS sequences in 3′ of the LTR and 5′ of the IRES. Primers were designed to amplify GFP from pMigR1. The forward primer included 22 nucleotides complementary to the 5′ end of GFP (5′- ATGGTGAGCAAGGGCGAGGAGC-3′) preceded in 5′ with an overhang (5′-CCACC3-′) to form a kozak sequence (CCACCATGGT), a 23-RSS (see Table 1) and a full *Sma*I (5′-CCCGGG-3′) restriction site. The reverse primer included 23 nucleotides complementary to the 3′ end of GFP (5′-TTACTTGTACAGCTCGTCCATGC-3′) followed by an overhang containing a 12-RSS and a full *Xho*I (5′-CTCGAG-3′) restriction site. The *Xho*I-*Sma*I digested PCR product was inserted into the *Xho*I-*Hpa*I digested MSCV-IRES-RFP vector, placing the GFP sequence in an inverted orientation relative to the 5′LTR.

**Table 1:** **RSSs tested with the GFPi-mRFP variants**.

Designation	Origin	RSS		
		Heptamer	Spacer	Nonamer
Con23^a^	IgKJ1^c,d^	CACAGTG	GTAGTACTCCACTGTCTGGCTGT	ACAAAAACC
ConPI	Synthetic^e^	CACAGTG	TTGGAACCACATCGGGAGCCTGT	ACAAAAACC
MI	Synthetic^e^	CACAGTG	TTG 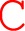 AACCACATC 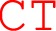 GAG 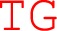 TGT	ACAAAAACC
MI-conPI(4)	Synthetic^e^	CACAGTG	TTGGAACCACATC 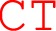 GAG 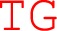 TGT	ACAAAAACC
MI-conPI(14,15)	Synthetic^e^	CACAGTG	TTG 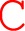 AACCACATCGGGAG 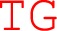 TGT	ACAAAAACC
MI-conPI(19,20)	Synthetic^e^	CACAGTG	TTG 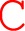 AACCACATC 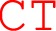 GAGCCTGT	ACAAAAACC
MI-CAGnon	IgHV1-63*02^c, d^	CACAGTG	TTG 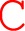 AACCACATC 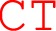 GAG 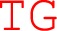 TGT	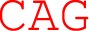 AAA 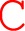 CC
Con12^b^	IgKV13-57*02^c,d^	CACAGTG	CTACAGACTGGA	ACAAAAACC
Jβ2-2	Jβ2-2^c,d^	CACAGTC	GTCGAAATGCTG	GCACAAACC
LMO2	cRSS^f,g^	aacaca – CACAGTA^i^	TTGTCTTACCCA	GCAATAATT
SCL	cRSS^d,h^	accaac – CACAGCC^i^	TCGCGCATTTCT	GTATATTGC

*^a^Con23 was paired with each 12-RSS variants*.

*^b^Con12 was paired with each 23-RSS variants*.

*^c^Mus musculus*.

*^d^International ImMunoGeneTics information system (http://www.imgt.org)*.

*^e^Cowell et al. (2004)*.

*^f^Homo sapiens*.

*^g^Dik et al. (2007)*.

*^h^Raghavan et al. (2001)*.

*^i^Small italic letters are 6 adjacent nucleotides in 5′ of the putative cRSS as found at the locus*.

GFPi-Cerulean Fluorescent Protein (CFP) (Figure 6), a second version of the GFPi reporter, bears the CFP instead of the mRFP and was optimized for medium throughput cloning of RSSs. The CFP sequence was amplified from pBS10 (Rizzo et al., 2004) using a 3′ primer containing a *Sal*I restriction site. The PCR product (*Sal*I-blunt) replaced the *Nco*I(blunt)-*Sal*I fragment containing mRFP in MSCV-IRES-RFP. The GFP inverted sequence was cloned as described above but using *Xho*I and *Hpa*I restriction sites in the 5′ and 3′ primers, respectively, without added RSS. This strategy allowed insertion of oriented sequences in 5′ or 3′ of GFP by ligation to the GFPi-CFP vector submitted to either a *Bgl*II and *Xho*I or *Hpa*I and *Eco*RI double digestion, respectively. Each inserted sequence was made of a pair of single-strand oligonucleotides (forward and reverse sequences) containing the RSS under test, three additional guanosines 5′ of the heptamer to improve RAG-mediated DNA DSB (Yu and Lieber, 1999) flanked by a *Bgl*II and *Xho*I or *Hpa*I and *Eco*RI restriction sites (Table 2). Upon phosporylation of each strand separately (1 μg), annealing was allowed along a temperature gradient from 80°C to RT.

**Table 2:** **Oligonucleotides used to construct the GFPi-CFP variants**.

Designation	Origin	Extension^f^	Heptamer	Sequence spacer	Nonamer	Extension^f^
Con23	IgKJ1^b^	5′-AACGGG	CACAGTG	GTAGTACTCCACTGTCTGGCTGT	ACAAAAACC	G-3′
3′^a^		3′-TTGCCC	GTGTCAC	CATCATGAGGTGACAGACCGACA	TGTTTTTGG	CTTAA-5′
MI-conPI(19,20)	Synthetic^c^	5′-AACGGG	CACAGTG	TTGCAACCACATCCTGAGCCTGT	ACAAAAACC	G-3′
3′		3′-TTGCCC	GTGTCAC	AACGTTGGTGTAGGACTCGGACA	TGTTTTTGG	CTTAA-5′
Con12	IgKV13-57*02^b^	5′-GATCTCGGG	CACAGTG	CTACAGACTGGA	ACAAAAACC	C-3′
5′		3′-AGCCC	GTGTCAC	GATGTCTGACCT	TGTTTTTGG	GAGCT-5′
Wu-Con12	Synthetic^d^	5′-AACGGG	CACAGTG	ATACAGACCTTA	ACAAAAACC	G-3′
3′		3′-TTGCCC	GTGTCAC	TATGTCTGGAAT	TGTTTTTGG	CTTAA-5′
LMO2	cRSS^e^	5′-AACGGG	CACAGTA	TTGTCTTACCCA	GCAATAATT	G-3′
3′		3′-TTGCCC	GTGTCAT	AACAGAATGGGT	CGTTATTAA	CTTAA-5′
3′Dd3	IgKJ1^e^	5′-GATCTCGGG	CACAGTG	GTAGTACTCCACTGTCTGGCTGT	ACAAAAACC	C-3′
5′		3′-AGCCC	GTGTCAC	CATCATGAGGTGACAGACCGACA	TGTTTTTGG	GAGCT-5′

*^a^The marks 3′ or 5′ indicate the position of the respective RSS in GFPi-CFP, relative to the inverted GFP sequence*.

*^b^International ImMunoGeneTics information system (http://www.imgt.org)*.

*^c^Cowell et al. (2004)*.

*^d^Ramsden et al. (1994)*.

*^e^Dik et al. (2007)*.

*^f^Extensions are cloning site + GGG when linked to the heptamer or cloning site only when linked to the non-amer*.

Plasmid DNA was prepared using a Miniprep column (Qiagen #12125) and proper inserts confirmed by sequencing. T4 polynucleotide kinase, T4 DNA ligase and Poly Ethylen Glycol were from Fermentas, restriction enzymes from New England Biolabs and oligonucleotides from Sigma-Aldrich.

Recombination signal sequences and primer sequences used to generate each GFPi variant are detailed in Tables 1 and 2 for GFPi-mRFP and GFPi-CFP, respectively.

### Cell culture

Human embryonic kidney 293T cells were cultured in DMEM medium (Invitrogen) supplemented with 10% Fetal Calf Serum (FCS), 100 U/mL penicillin and streptomycin (Invitrogen), at a temperature of 37°C and 5% CO_2_ conditions and re-seeded at low density every 2–3 days. Reh, NALM-6, SUP-T1, Jurkat, HL-60, and K-562 cell lines were cultured in RPMI Glutamax medium (Invitrogen) supplemented with 10% FCS, 100 U/mL penicillin and streptomycin, and 0.55 mM β-mercaptoethanol, at 37°C and 5% CO_2_ and kept at a density of 0.25 × 10^6^ cells/mL. Cultures enriched in B cell progenitors were established as described in Carvalho et al. (2001) from bone marrow cell suspensions prepared by flushing femurs and tibias with a 23-gage needle and grown at the density of 1 × 10^6^ cells/mL in Optimem supplemented with 10% FCS, 100 U/mL penicillin and streptomycin, 0.55 mM β-mercaptoethanol, and 10 ng/mL murine recombinant IL-7 (PeproTech).

### GFPi based *in vitro* recombination assay

**Figure 3:**
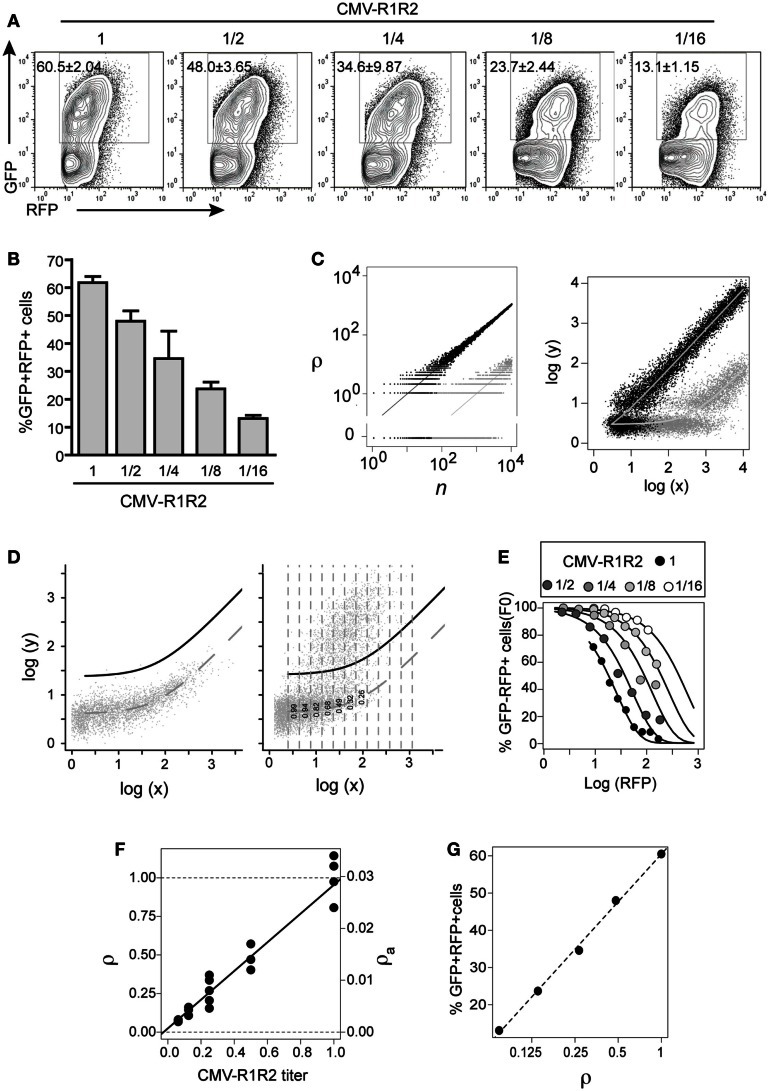
**GFPi predictably quantifies RAG activity**. The 12- and 23-consensus RSS GFPi was co-transfected with serial 2-fold titration of CMV-RAG1/2 constructs (from 1.6/1.4 to 0.2/0.175 μg, final dilution 1/16th of the initial plasmid DNA amount). **(A)** Representative plots of each RAG expression condition. **(B)** Recombination efficiency defined by% of GFP^+^ cells inside an RFP^+^ gate (test – background) with means and standard deviations of at least three replicates. **(C)** Relationship between the number of recombined plasmids *r* in cells containing *n* total plasmids (left) and of the corresponding GFP and RFP values (right). The theoretical model assumes *r* is Poisson distributed with mean ρn, where ρ is the recombination coefficient. Left panel: the dots represent two random realizations of the model for ρ = 0.1 (black) and ρ = 0.001 (gray), while the lines represent the mean values. Right panel: the dots represent the logarithm of the RFP (*x*) and GFP (*y*) signals corresponding to the number of plasmids in the left panel, assuming that autofluorescence, amplification, variation and measurement noise result in bivariate normal-distributed around log-mean values (curves). **(D)** Estimation of apparent recombination coefficient ρ_a_ in GFPi assays by obtaining the frequency of cells with zero recombination events in binned RFP data (left and center). Left panel: experimental bivariate GFP vs. RFP reporter log-intensities measured in cells transfected with GFPi alone (without RAG) were fitted by the model log(y) = log(*x*_0_ + *bx*), by least square minimization and minimization of residuals mean and trend. The dashed curve represents the best fit and the full black curve represents the best fit added by four times standard deviation of the residuals. Right panel: experimental bivariate GFP vs. RFP log-intensity measurements in cells co-transfected with RAG were binned according RFP log-intensity and the frequency of GFP^−^ cells in each bin scored (i.e., with GFP values below the black curve). **(E)** Fitting the model P(r = 0) = exp(−ρ_a_(x − x_0_)) to the experimental data. The frequency of GFP^−^ cells was plotted vs. the mean RFP log-intensity per bin for the cells with serial dilutions of CMV-RAG1/2 constructs **(A)**, and fitted by the model. **(F)** The relative and apparent recombination coefficients (respectively ρ and ρ_a_) in RAG titration experiments are proportional to the expected RAG activity. The estimates of ρ_a_ obtained by model fitting (as in **(D)** were normalized such that the relative recombination coefficient ρ at the highest titer is 1. Both sets of value are plotted as a function of the CMV-RAG1/2 titers, ρ_*a*_ = 0.0007 + 0.028[CMV-R1R2], ρ = 0.02 + 0.93[CMV-R1R2]. **(G)** The frequency of GFP^+^ cells inside the RFP^+^ gate (as in **B**) is proportional to the logarithm of the relative recombination coefficient ρ.

293T were seeded at a density of 0.5 × 10^6^ cells per 6-well plate well in 2.5 mL of medium, co-transfected 24 h after by replacing 600 μL of medium by a transfection mix containing 10 μL of Lipofectamine2000 (Invitrogen), 600 μL of Serum-free Optimem and a total of 10 μg of plasmid DNA: 5 μg of GFPi and 5 μg of mock plasmid (negative control); or 2.5 μg of H2k-RAG1 and 2.5 μg of H2k-RAG2; or 2.5 μg of H2k-RAG1 and 2.5 μg of H2k-RAG2ER^TAM^, or equimolar amounts of CMV-RAG1 (1.6 μg) and CMV-RAG2 (1.4 μg), unless otherwise indicated (Figures 2 and 3). After 16 h incubation, cells were washed, incubated for 48 h with fresh medium and harvested for flow cytometry analysis of GFP and RFP or CFP fluorescence. In assays carried out with H2k-RAG2ER^TAM^, medium was supplemented either with vehicle (2:10000 ethanol, Sigma) or 200 nM 4-hydroxytamoxifen (4-OHT, Sigma).

### Flow cytometry analyses

For the GFPi-mRFP assays, flow cytometry data were acquired with a MoFlo (Dako Cytomation-Beckman Coulter). RFP was excited with a 561-nm CrystaLaser GCL-050-561 50 mW DPSS laser coupled to fiber optics (38 mW output) and emission detected using a 630/75-nm bandpass filter on channel 7 (FL7); GFP was excited with a 488-nm Coherent Sapphire 488-200 CDRH (140 mW output) and emission detected using a 530/40-nm filter pass on channel 1 (FL1). In some cases, experiments were reproduced with data acquired with a BD FACSAria III: RFP was measured on the PE-Texas Red/mCherry channel, excited with a Yellow Green 561 nm Laser and emission detected with a 610/20-nm filter pass; GFP was measured on the FITC channel, excited with a 488-nm Blue Laser and emission detected with a 530/30-nm filter pass.

For the GFPi-CFP assay, data were acquired on a CyAn ADP (Beckman Coulter), a BD FACSAria III or on a LSR Fortessa (BD Bioscience), using a 96well-plate auto sampler. GFP was excited and detected using the same wavelengths as described above for the MoFlo when using the CyAn ADP or as described for the FACSAria III when using the LSR Fortessa analyzer. The CFP was either excited by a 405-nm laser using a 450/50-nm filter for detection or by a 442-nm Blue-Violet laser and measured using a 470/20-nm filter pass (LSR Fortessa) which considerably increased the mean fluorescence intensity of the CFP. Data were collected with the following programs: FACSDiva (BD Bioscience) for the LSR Fortessa and FACSAriaIII, Summit (Beckman Coulter) for the Cyan ADP and the MoFlo. In all cases, data analysis was performed using the FlowJo software (Tree Star Inc.).

### PCR and sequence analyses of recombined GFPi

Plasmid DNA was recovered using the Qiaprep Spin Miniprep Kit (Qiagen) from GFP^−^/RFP^+^ or GFP^+^/RFP^+^ cell populations sorted from 293T cells previously co-transfected with GFPi and mock DNA or GFPi and CMV-RAG plasmids, respectively. PCR reactions were performed using the indicated amounts of recovered plasmids as template and the following primers (depicted in Figure 2): forward – 5′-AGCCCTTTGTACACCCTAAG-3′ and reverse – 5′-GTTGTACTCCAGCTTGTGCC-3′ to amplify the coding joint (CJ) region of rearranged GFPi (GFPi-RR) plasmids; forward – 5′-CATCTTCTTCAAGGACGACGG-3′ and reverse – 5′-CGGCCAGTAACGTTAGG-3′ to amplify the signal joints (SJ) of GFPi-RR plasmids; and, forward – 5′-AGCCCTTTGTACACCCTAAG-3′ and reverse – 5′-CATCTTCTTCAAGGACGACGG-3′ to detect unrearranged GFPi (GFPi-UNR). To sequence CJ and SJ, the respective PCR products were separated in an agarose gel and fragments from the expected size extracted using the Gel Extraction Kit (Zymo), cloned into the pGEM-T Easy Vector (Promega) and sequenced with the T7 primer (5′-TAATACGACTCACTATAGG-3′) and the SP6 primer (5′-ATTTAGGTGACACTATAG-3′).

### Viral production and transduction

Viral production and transduction were carried out as described in (Sarmento et al., 2005). Briefly, MSCV-IRES-RFP or GFPi were co-transfected into 293T cells using a calcium phosphate precipitation method together with the amphotropic packaging plasmid pKat and the pCMV-VSV-G plasmid encoding the vesicular stomatitis virus G-glycoprotein. Supernatants containing pseudo-typed retrovirus were collected at 48 and 72 h post-transfection and passed through a 0.45-μm filter. Transduction of Reh, NALM-6, SUP-T1, Jurkat, HL-60, and K-562 cell lines as well as mouse bone marrow cells cultured for 1 week in the presence of IL-7 was carried out with 1–2 × 10^5^ cells resuspended in 500 μL of viral supernatant and 500 μL of medium supplemented with Polybrene (Sigma) to a final concentration of 4 μg/mL, followed by spinoculation of 90 min, 37°C at 2200 rpm and incubation for the indicated time.

### Bone marrow transduction and transplantation

Retroviral transduction of bone marrow cells and transfer into lethally irradiated recipients was adapted from (Pear et al., 1996). Bone marrow cells were collected from 6- to 12-week-old C57BL/6 or RAG2 knock-out mice 4 days after intravenous administration of 250 mg/kg of fluorouracil (5-FU, Mayne Pharma). The cells were cultured overnight in IMDM supplemented with 15% FCS, 100 U/mL of penicillin and streptomycin in the presence of the following cytokines (PeproTech): 10 ng/mL IL-3, 5 ng/mL IL-6, and 100 ng/mL SCF. The cells were then washed, resuspended in a mixture with half volume of viral supernatant and half volume of culture medium, supplemented with the same cytokine cocktail and polybrene (4 μg/mL, Sigma), and centrifuged at 2200 rpm, 37°C for 90 min. A second round of spinoculation was performed the following day. After washing with PBS, at least 5 × 10^5^ cells were injected intravenously into lethally irradiated (9 Gy) C57BL/6 recipients. Mice were bled 7 weeks post-transfer and peripheral blood cells were analyzed by flow cytometry. All experiments were performed in accordance with the guidelines for the care and use of animals under an animal protocol approved by the Instituto Gulbenkian de Ciência Animal Care and Use Committee.

### Mathematical modeling and statistical analyses

The frequency of GFP^+^ cells inside the RFP^+^ gate provides a straightforward empirical measurement of the recombination efficiency (RE). The corresponding values are reported for each *in vitro* recombination assay as an average and a standard deviation of at least three replicates from at least two independent experiments. Whenever the average frequency of double-positive events observed in the mock/GFPi control (no RAG) is not shown, the RE of each GFPi variant in the presence of RAG activity is calculated by subtracting the mock background values. The statistical significance in all pair wise comparison of relative frequencies of GFP^+^ were performed using the Student’s *t*-test, applying Welch correction when necessary, and correcting the significance level for multiple comparison.

The frequency of GFP^+^ cells is related to the intensity of GFP and RFP signal in each cell using a mathematical that assumes multiple reporter plasmids. We define the recombination coefficient ρ as the probability that each plasmid is independently recombined in the time of the assay. Considering that this probability is relatively low, we assume that the number of recombined plasmids *r* in a cell containing *n* total plasmids is approximately Poisson distributed with mean ρ*n* (illustrated in Figure 3C-left). We are particularly interested in the probability that none of the *n* plasmids is recombined which is *P*(*r* = 0|*n*) = *exp*(-ρ*n*). The expected intensity of the default reporter signal (i.e., RFP) in cells containing n total reporter plasmids is *x*_0 _+ *m_x_* *n*, where *x*_0_ is the mean basal signal and *m_x_* is the mean signal due to a single plasmid. Likewise, the expected intensity of the recombination-associated signal (i.e., GFP) in cells containing *r* recombined reporter plasmids is *y*_0 _+ *m_y_ r*, where *y*_0_ is the mean basal signal and *m_y_* is the mean signal due to a single recombined plasmid. In practice, random variation expression of two genes of the reporter plasmid and measurement noise leads to a distribution of the measured signals around these expected values (illustrated in Figure 3C-right). From the linear relationship between the signal *x* and the number of total plasmids one obtains *n* = (*x − x*_0_)/*m_x_*, which allows us to obtain an expression for the probability that the cells did not recombine any of its *n* reporter plasmids as a function of the measured *x*-signal intensity: *P*(*r* = 0*|n*) = *exp*(*−*ρ(*x − x*_0_)/*m_x_*) = *exp*(*−*ρ*_a_*(*x − x*_0_), where ρ*_a_* is an apparent recombination coefficient proportional to the true recombination coefficient ρ with proportionality constant 1/*m_x_*. The apparent recombination coefficient ρ*_a_* is estimated by fitting the expression to empirical data of the relative frequency of cells without recombined plasmids, denoted *F*_0_(*x*), as a function of the *x*-signal intensity, i.e., the relative frequency of GFP^−^ cells as a function of intensity of RFP signal (illustrated in Figure 3F).

The data processing, model fitting and estimation were performed in R. Briefly, bivariate (RFP signal, GFP signal) data were exported from FlowJo as scaled values after appropriate gating and subsequently imported into the statistical software R. The function log_10_(*y*) = log_10_(*x*_0_ + *b*_0x_) is fitted to data from samples without nominal RAG activity by minimizing least-squares and ensuring that the residuals have mean zero and no trends, and the standard deviation of the residuals *S*_0_ computed. The relative frequency of cells without recombined plasmids in any sample of interest is obtained by binning on the *x*-axis, and computing within each bin the frequency of cells fulfilling the condition *log*_10_(*GFP*) ≤ *log*_10_(*RFP*_0 _+ *b*_0RFP_) + *kS*_0_, where *k* is an appropriate constant. This procedure avoids problems with color compensation and works directly with uncompensated data. The recombination coefficient associated to each sample is obtained by fitting the model *P*(*r* = 0) = *exp*(−ρ*_a_*(*x* − *x*_0_)) to the binned frequencies by non-linear least square. Analysis of simulated data indicated that 11 bins lead to robust estimates and that more accurate and precise estimates are obtained if bins with less than 5% of all the gated events are neglected. Furthermore, *k* is fixed such that the binned frequencies in samples without RAG are over 99.9% (typically in the range 3–5). This tool is being further developed for adaptation to a user-friendly platform and is available upon request.

The frequency of GFP^+^ inside the RFP^+^ gate, which is used as an empirical proxy of the RE throughout this article, is proportional to the logarithm of the recombination coefficient (Figure 3G).

## Results

### GFPi is a RAG-dependent recombination reporter

We have engineered the reporter for RAG1/2 activity (GFPi) in the MSCV-IRES-RFP retroviral vector backbone that allows the detection of the substrate, revealed by the IRES-driven expression of the RFP. Whenever RAG-dependent recombination events occur, the 12-RSS and 23-RSS sequences are targeted by RAG, leading to the inversion of the Green Fluorescent Protein (GFP) coding sequence and therefore rendering coupled RFP and GFP expression (Figure 2A).

To assess the functionality of our reporter, 293T cells were transfected with GFPi containing consensus 12 and 23-RSS either alone or together with equimolar amounts of CMV-RAG1 and 2 (Figure 2B). The transfection efficiency was routinely ≥50%, as revealed by the percentage of RFP^+^ cells. In absence of RAG the background GFP signal was always <1% while in presence of RAG a RFP^+^GFP^+^ cell population was readily detectable. The frequency of transfected cells that underwent recombination is determined by the percentage of GFP^+^ cells inside an RFP^+^ gate, to which value one can subtract the background defined in absence of RAG.

We next confirmed that the GFP^+^ cells carried RAG-dependent rearranged GFPi (Figure 2C). Double-positive RFP^+^GFP^+^ cells from cells co-transfected with GFPi and RAG were sort purified, the plasmid DNA recovered and tested for the presence of recombination-specific amplicons by semi-quantitative PCR. Plasmid DNA extracted from RFP^+^ cells from cultures transfected with GFPi only served as control. Amplification of signal and CJs resulting from GFPi inversion was readily detectable in the plasmid preparations recovered from RFP^+^GFP^+^ cells but not in control RFP^+^ cells. As each cell received several copies of the reporter upon transfection, GFPi-UNR amplicons were detectable in both cell populations. The rearranged GFPi was further confirmed to display signature of RAG-dependent recombination by sequencing the PCR products (Tables 3 and 4). As expected, CJs presented end-processing by nucleotide excision and addition as well as palindromic sequences (P nucleotides) at the position of the repaired junction; N nucleotides were not observed nor expected as TdT is not expressed in non-lymphoid cells (Komori et al., 1993). The processing of the coding joints occasionally affected the GFP sequence, removing one or two nucleotides, an imperfection we corrected in the subsequent version of the GFPi vector (see below and Figure 7). Nevertheless, as upon transfection each cell bears several copies of GFPi, impaired expression of the marker from one or few rearranged molecules per cell would not significantly affect the readout. Finally, and as expected, SJ were the product of the direct ligation of blunt signal ends. We conclude that the GFPi assay reports RAG activity.

**Table 3:** **Sequences of rearranged GFPi-mRFP Con12/Con23 coding joints**.

Clones	5′ – Coding end			Insertions	3′ – Coding end	
	pMSCV5′	*Bgl*I	*Xho*I		Kozak	5′GFP
Germline	CTTCTCTAGGCGCCGGAATT	AGATCT	CTCGAG		CCACC	ATGGTGAGC
CJ1	CTTCTCTAGGCGCCGGAATT	AGATCT	CTC- - -		- - - - -	-TGGTGAGC
CJ2	CTTCTCTAGGCGCCGGAATT	AGATCT	CTCGA-	C	- - -CC	ATGGTGAGC
CJ3	CTTCTCTAGGCGCCGGAATT	AGATCT	CTCGAG	CT	- - - - -	ATGGTGAGC
CJ4	CTTCTCTAGGCGCCGGAATT	AGATCT	CTCGAG		- - - -C	ATGGTGAGC
CJ5	CTTCTCTAGGCGCCGGAATT	AGATCT	CTCG- -		- - -CC	ATGGTGAGC
CJ6	CTTCTCTAGGCGCCGGAATT	AGAT- -	- - - - - -	TTTTGGG	CCACC	ATGGTTGCC
CJ7	CTTCTCTAGGCGCCGGAATT	AGATCT	CTC- - -		- - - - -	ATGGTGAGC
CJ8	CTTCTCTAGGCGCCGGAATT	AGATCT	CTCG- -		CCACC	ATGGTGAGC
CJ9	CTTCTCTAGGCGCCGGAATT	AGATCT	CTCGAG		- - - - -	- -GGTGAGC
CJ10	CTTCTCTAGGCGCCGGAATT	AGATCT	CTCGAG		- - - -C	ATGGTGAGC
CJ11	CTTCTCTAGGCGCCGGAATT	AGATCT	CTCG- -		- - - - -	-TGGTGAGC
CJ12	CTTCTCTAGGCGCCGGAATT	AGATCT	CTCGAG	C	- - - - -	-TGGTGAGC
CJ13	CTTCTCTAGGCGCCGGAATT	AGATCT	CT- - - -		- - - -C	ATGGTGAGC
CJ14	CTTCTCTAGGCGCCGGAATT	AGATCT	CTCGA-		- - - - -	- -GGTGAGC
CJ15	CTTCTCTAGGCGCCGGAATT	AGATCT	CTCGA-	TGGC	CCACC	ATGGTGAGC
CJ16	CTTCTCTAGGCGCCGGAATT	AGATCT	CTCG- -		- - - - -	-TGGTGAGC
CJ17	CTTCTCTAGGCGCCGGAATT	AGATCT	CTCGAG		- - - -C	ATGGTGAGC
CJ18	CTTCTCTAGGCGCCGGAATT	AGATCT	CTCGAG		- - - -C	ATGGTGAGC
CJ19	CTTCTCTAGGCGCCGGAATT	AGATCT	CTCG- -	G	CCACC	ATGGTGAGC
CJ20	CTTCTCTAGGCGCCGGAATT	AGATCT	CTCG- -		- - - - -	-TGGTGAGC
CJ21	CTTCTCTAGGCGCCGG- - - -	- - - - - -	- - - - - -		- - - -C	ATGGTGAGC
CJ22	CTTCTCTAGGCGCCGGAATT	AGATCT	CTCGAG	CT	- - - - -	-TGGAGAGC
CJ23	CTTCTCTAGGCGCCGGAATT	AGATCT	CTCGA-		- - - -C	ATGGTGAGC
CJ24	CTTCTCTAGGCGCCGGAATT	AGATCT	CTCGAG		- - - -C	ATGGTGAGC

*Deleted nucleotides are represented by dashes and potential *P* elements marked in italics*.

**Table 4:** **Sequences of rearranged GFPi-mRFP Con12/Con23 SJ**.

Clones	12-RSS			23-RSS		
	Nonamer	Spacer	Heptamer	Heptamer	Spacer	Nonamer
Germline	GGTTTTTGT	TCCAGTCTGTAG	CACTGTG	CACAGTG	CTACAGCTCCACTGTCTACTGGA	ACAAAAACC
SJ1	GGTTTTTGT	TCCAGTCTGTAG	CACTGTG	CACAGTG	CTACAGCTCCACTGTCTACTGGA	ACAAAAACC
SJ2	GGTTTTTGT	TCCAGTCTGTAG	CACTGTG	CACAGTG	CTACAGCTCCACTGTCTACTGGA	ACAAAAACC
SJ3	GGTTTTTGT	TCCAGTCTGTAG	CACTGTG	CACAGTG	CTACAGCTCCACTGTCTACTGGA	ACAAAAACC
SJ4	GGTTTTTGT	TCCAGTCTGTAG	CACTGTG	CACAGTG	CTACAGCTCCACTGTCTACTGGA	ACAAAAACC
SJ5	GGTTTTTGT	TCCAGTCTGTAG	CACTGTG	CACAGTG	CTACAGCTCCACTGTCTACTGGA	ACAAAAACC
SJ6	GGTTTTTGT	TCCAGTCTGTAG	CACTGTG	CACAGTG	CTACAGCTCCACTGTCTACTGGA	ACAAAAACC
SJ7	GGTTTTTGT	TCCAGTCTGTAG	CACTGTG	CACAGTG	CTACAGCTCCACTGTCTACTGGA	ACAAAAACC
SJ8	GGTTTTTGT	TCCAGTCTGTAG	CACTGTG	CACAGTG	CTACAGCTCCACTGTCTACTGGA	ACAAAAACC
SJ9	GGTTTTTGT	TCCAGTCTGTAG	CACTGTG	CACAGTG	CTACAGCTCCACTGTCTACTGGA	ACAAAAACC
SJ10	GGTTTTTGT	TCCAGTCTGTAG	CACTGTG	CACAGTG	CTACAGCTCCACTGTCTACTGGA	ACAAAAACC
SJ11	GGTTTTTGT	TCCAGTCTGTAG	CACTGTG	CACAGTG	CTACAGCTCCACTGTCTACTGGA	ACAAAAACC
SJ12	GGTTTTTGT	TCCAGTCTGTAG	CACTGTG	CACAGTG	CTACAGCTCCACTGTCTACTGGA	ACAAAAACC
SJ13	GGTTTTTGT	TCCAGTCTGTAG	CACTGTG	CACAGTG	CTACAGCTCCACTGTCTACTGGA	ACAAAAACC
SJ14	GGTTTTTGT	TCCAGTCTGTAG	CACTGTG	CACAGTG	CTACAGCTCCACTGTCTACTGGA	ACAAAAACC
SJ15	GGTTTTTGT	TCCAGTCTGTAG	CACTGTG	CACAGTG	CTACAGCTCCACTGTCTACTGGA	ACAAAAACC

### GFPi assay responds to RAG activity titration in a predictive manner

We next assessed the quantitative response of the GFPi assay to controlled variations in recombination activity. To this end, we titrated down CMV-RAG1/2 plasmids in the transfection mixture by serial twofold dilutions. As shown in Figure 3A, GFPi assay displays a clear dose-dependent response. From the highest to the lowest concentration of RAG expression vectors, the frequency of GFP^+^ cells measured by FACS progressively decreases from 60% to 13%. The low standard deviation we obtained across repeats and across experiments illustrate that variation in the amount of effectively transfected DNA is negligible. In a first approach we quantified the recombination activity in our assay by simply monitoring the percentage of GFP^+^ cells detected in the RFP^+^ population. This empirical RE score was calculated by subtracting the background values obtained in control cells transfected with GFPi in absence of RAG (Figure 3B).

However, it was conspicuous that the center of mass of the GFP^+^ cells progressively shifts to lower GFP values and higher RFP intensities, as the expected RAG activity decreases. To understand how both the frequency of GFP^+^ cells and the GFP intensity depend quantitatively on the underlying RE in the assay we developed a mathematical model. The model describes the number of recombined target episomes per cell as a function of the probability of recombining targets during the assay, that we call recombination coefficient ρ. We assume that the number of recombined targets is Poisson distributed with mean equal to the product of ρ by number of targets per cell. Two random realizations of the model for two values of the recombination coefficient (ρ = 0.1 and ρ = 0.01) are depicted in Figure 3C (left), illustrating the discreteness of the number of plasmids around the mean values depicted by the trend lines. The RFP and GFP signals measured by flow cytometry are random continuous variables proportional to the total and recombined plasmid numbers, respectively, which masks the underlying discreteness as illustrated in Figure 3C (right). In this model, as in the real data, when the recombination coefficient decreases the center of mass of the GFP^+^ cells in the bivariate space progressively shifts to lower GFP values and higher RFP values.

The key question then becomes how to infer the recombination coefficient from the experimental bivariate data obtained by flow cytometry? The model suggests two potential approaches. It would be tempting to use the asymptotic linear relationship for large values of reporter per cell, which is predicted in the model and observed in the data in the case of high recombination efficiencies (Figure 3A CMV-R1R2 high titers). However, simulations of the model (not shown) indicate that for low recombination efficiencies this strategy gives estimates that are not accurate. A second alternative to infer the recombination coefficient is to consider the probability that a cell with a given RFP signal intensity makes no recombination during the time of the assay. According to the model this probability is given by exp(−ρ_a_(x − x_0_)), where RFP intensity measured in the cell, x_0_ is the mean RFP autofluorescence, and ρ_a_ is an apparent recombination coefficient that is proportional to the true underlying recombination coefficient ρ, with the proportionality constant being the average RFP signal obtained from a single episome. By taking ratios between two values of ρ_a_ obtained in identical experimental settings the proportionality constant cancels and one can obtain the relative ratio between the true ρ values. Simulations of the model (not shown) indicated that accurate estimate of ρ_a_ and relative ρ values can be obtained by fitting the above probability as a function of RFP values to the relative frequencies of GFP negative cells scored at different log-intensities of RFP signal.

We used this method to infer the apparent and relative recombination coefficients underlying the data obtained for serial dilutions of CMV-RAG1/2 plasmid in Figure 3A. The following procedure was used to compute the frequencies of GFP negative cells for different values of RFP intensities. First, we fitted a linear model to the bivariate data obtained in cells not transfected with CMV-RAG1/2 plasmid (dashed curve in the plots Figure 3D), ensuring that the GFP log-intensity residuals of the fitting (i.e., the differences between observed GPF log-intensity and the expected values) had approximately null values of mean and regression coefficient against the RPF log-intensity (not shown). We then defined a cutoff curve by adding four to five standard deviations of the residuals to the fitted line (black curve in the plots Figure 3D). The number of standard deviations added to the mean value was set to ensure that the frequency of GFP negative cells (i.e., cells with GFP below the cutoff curve) measured in cells without RAG activity is unitary with a precision <10^−4^. For each cell population transfected with the different Rag plasmids titers we binned the data according to the RFP log-intensity and computed the frequencies in each bin (illustrated for the unitary CMV-R1/R2 titer in Figure 3D-right). Using the GFP negative frequencies per RFP signal intensity thus obtained for each data set (Figure 3E) we estimated the values of apparent RE (ρ_a_) by non-linear least square fitting of the theoretical probability (Figure 3F). As expected from mass action, the relative recombination coefficient ρ (ρ_a_ normalized by the mean value obtained for the CMV-R1/R2 higher titer) in cells transfected with CMV-R1/R2 plasmid increases linearly with the plasmids titer, and the regression line has a regression coefficient close to the unit and predicts approximately zero recombination at zero titer (Figure 3F). This means that the recombination coefficient estimated from the GFPi assay is an accurate quantitative measurement. Furthermore, the frequency of GFP^+^ cells within the RFP^+^ gate obtained directly from gating the flow cytometry data (as in Figure 3B) scales linearly with the logarithm of the relative recombination coefficient ρ (Figure 3G). In turn, this result indicates that this simple frequency, determined by classical FACS analysis, can be used as a quantitative proxy of the RE.

### GFPi quantifies RAG transcription and nuclear translocation

**Figure 4:**
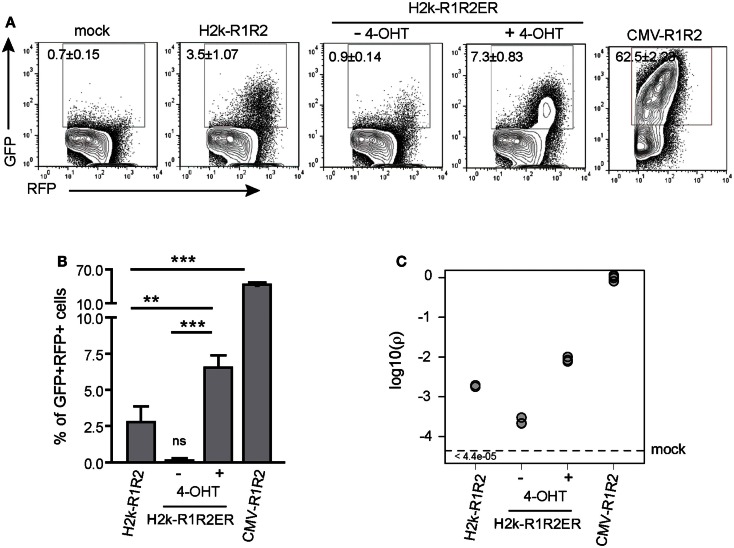
**GFPi reports RAG transcription and nuclear translocation efficiencies**. **(A)** Representative FACS analysis of 293T cells transfected with the GFPi reporter only (mock) or together with H2k-RAG1/2, H2k-RAG1 and H2k-RAG2ER^TAM^ treated with vehicle alone or with 4-OHT, or CMV-RAG1/2. **(B)** Recombination efficiency determined as [(%GFP^+^ in test) – (%GFP^+^ in mock)]. Mean and standard deviation of at least six replicates and at least two independent experiments. (** – *p* < 0.05, *** – *p* < 0.001, ns – not significant). **(C)** Relative recombination coefficient ρ when CMV-R1R2 is 1.

We next tested whether the GFPi assay detects quantitative differences in efficiency of RAG1/2 transcription or RAG2 nuclear translocation (Figure 4) Variation in transcription efficiency was achieved by transfection of RAG1/2 expression vectors bearing different promoters, namely H2k or CMV (see Figure 1). Variation in nuclear translocation efficiency was achieved by transfection of a construct that contains the RAG2 coding sequence fused to the ligand-binding domain of the estrogen receptor (see Figure 1). The gene is under the H2k promoter and the fusion protein translocates efficiently to the nucleus in presence of an estrogen analog. This construct was used to generate transgenic mice and confirmed to be able to complement the RAG2 knock-out lymphocyte deficiency *in vivo* specifically in the presence of the estrogen analog, 4-OHT (Sarmento and Bonnet, in preparation). The 293T cells were transfected with GFPi together with mock plasmid, H2k-RAG1/2, H2k-RAG1, and H2k-RAG2ER or else CMV-RAG1/2 (Figure 4A). The frequency of GFP^+^ cells inside the transfected RFP^+^ subset were dependent on the strength of the promoter driving RAG expression (3.5 ± 1.1% under H2k vs. 62.5 ± 2.2% under CMV) and of the nuclear translocation of RAG2 (0.9 ± 0.1% upon vehicle alone vs. 7.3 ± 0.8% upon 4-OHT induction) (Figure 4B). The estimated recombination coefficient ρ spans about four orders of magnitude in these different setting (Figure 4C). As an example of an application of the GFPi assay, this experiment indicated that the RAG2ER construct present some degree of leakiness when transfected into untreated 293T. The flow cytometry profiles of the mock (no RAG) and the test (H2k-R1R2ER no 4-OHT) are different with noticeable GFP^+^ signal in the latter, absent in the former. This difference was not quantifiable by simple percentage analysis but revealed by a relative recombination coefficient ρ of 2.3 × 10^−4^ ± 0.5 × 10^−4^, close but above the minimum value detectable in the controls (5 × 10^−5^). This case also illustrates that when RAG is limiting, only cells with high RFP expression levels, hence with high copy number of GFPi, rearrange enough GFPi to emit a detectable signal. Monitoring GFP^+^ frequency in the last percentiles of RFP intensity also enhances the sensitivity of the analysis, although this approach is more easily confounded by variation in the flow cytometry analyzer settings (not shown).

Finally, we evaluated by western blot analysis that RAG1/2 expression was about 10-fold increased when driven by CMV instead of H2k, after equimolar transfection (data not shown). Analysis of the data obtained with the GFPi assay reported a 15-fold difference in the recombination coefficient ρ between H2k and CMV-driven RAG. Together, these results demonstrate that GFPi, when used as an episomal substrate, provides a faithful quantitative assay to detect recombination driven by ectopic RAG1/2 activity.

### GFPi functions as an integrated substrate *ex vivo* and *in vivo*

**Figure 5:**
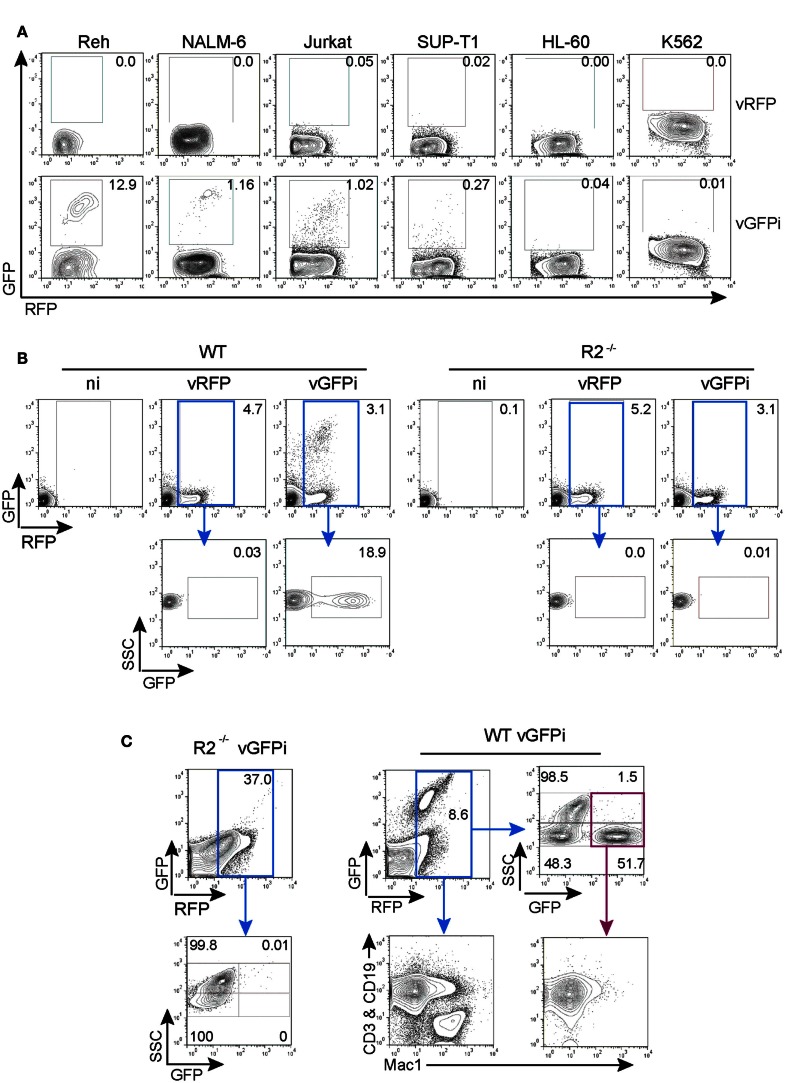
**GFPi retrovirus is a RAG1/2-specific reporter substrate *ex vivo* and *in vivo***. **(A)** Human lymphoid leukemic cell lines of B (Reh, NALM-6) and T (Jurkat, SUP-T1) cell origin as well as myeloid leukemia and erythroleukemia cells lines (HL-60 and K-562, respectively) were transduced with MSCV-IRES-RFP (vRFP) or vGFPi retrovirus. Frequency of cells that underwent RAG recombination was measured by FACS 1 week post-transduction. **(B)** Bone marrow cells of wild-type (WT, left) or RAG2 knock-out (R2^−/−^, right) C57BL/6 mice cultured in the presence of IL-7 and either not infected (ni) or infected with MSCV-IRES-RFP (vRFP) or GFPi reporter (vGFPi) virus were analyzed by FACS for the expression of RFP and GFP to detect RAG-mediated recombination 1 week post-transduction. The plots are representative of three independent experiments. **(C)** Lethally irradiated C57BL/6 mice were reconstituted with BM cells isolated from 5-FU-treated R2^−/−^ or WT C57BL/6 mice after transduction with the GFPi or the RFP retrovirus. Shown are representative contour plots (*n* = 3 for each type of chimeras) of peripheral blood cells analyzed by FACS 7 weeks post-transfer. A first gate, not shown, identified the mononuclear cell population according to the forward and side-scatter physical parameters. Cells were then analyzed for RFP and GFP expression, gated on RFP^+^ cells (blue gate) and reanalyzed for SSC and GFP. The SSC^high^ subset is enriched in myeloid cells, while the SSC^low^ population are enriched in lymphoid cells. For WT cells, RFP^+^ cells were also analyzed for the distribution of lymphocytes (CD3^+^ or CD19^+^) by co-staining in the same channel (PE) vs. macrophages/myeloid cells (MAC1/CD11c^+^). Analysis of GFP^bright^RFP^+^ (purple gate) for the same lineage markers documents rearrangement in lymphocytes.

We next tested whether integrated GFPi substrates delivered through retroviral infection would specifically recombine in a RAG-dependent manner (Figure 5). We first selected five hematopoietic leukemia cell lines for which RAG1/2 activity has been previously quantified using a classical episomal substrate and readout of recombination by differential antibiotic resistance of subsequently transfected bacteria (Gauss et al., 1998). In this early work the Reh and NALM-6, two B cell acute lymphoid leukemia phenotypically resembling a pre-B developmental stage, displayed efficiency of recombination evaluated as 21.6 and 1.8% respectively; the SUP-T1 non-Hodgkin’s lymphoma, double-positive for CD4 and CD8, scored as 0.09% while the acute myelogenous leukemia HL-60 and the chronic myelogenous leukemia K-562 scored at 0.008 and 0.02% respectively (Gauss et al., 1998). We also tested the Jurkat acute T cell leukemia cells that express a mature TCR and low levels of Rag mRNAs (Roose et al., 2003). These tumor cells were infected with control MSCV-IRES-RFP or GFPi retrovirus (Figure 5A). All four lymphoid cell lines (Reh, NALM-6, Jurkat, and SUP-T1) but not the myeloid tumors presented a RFP^+^GFP^+^ population when infected with GFPi, confirming the ability of the GFPi retrovirus to detect endogenous RAG1/2 activity. Defining the RE by simple percentage analysis for each cell type allowed their ranking for RAG activity, in concordance with that established earlier, although we did not detect the residual RAG1/2 activity reported to occur in the leukemias of myeloid lineage HL-60 and K-562 (Gauss et al., 1998). Each cell line presents specific size, volume, granularity, and consequently autofluorescence. Moreover each cell line may differently express the DNA binding factors allowing MSCV driven gene expression. With these uncontrolled variations, a formal quantitative assessment of the specific RAG activity with GFPi is unwarranted.

We next assessed whether GFPi reveals RAG activity in primary cells *ex vivo*. Mouse bone marrow cells from WT and RAG2^−/−^ animals grown in the presence of IL-7 to support the proliferation and survival of B cell progenitors were infected with control MSCV-IRES-RFP or GFPi retrovirus (Figure 5B). FACS analysis revealed the appearance of a double-positive RFP/GFP population exclusively in the GFPi-infected WT cells.

To test the applicability of the GFPi reporter *in vivo*, bone marrow progenitor cells isolated from WT and RAG2^−/−^ mice infected with GFPi were transferred into lethally irradiated WT recipient mice (Figure 5C). RFP positive cells were readily detected 7 weeks post-transplant in the peripheral blood of all mice and GFP^+^RFP^+^ cells were detected solely in mice reconstituted with WT GFPi-infected cells, restricted to the side-scatter low lymphocyte-enriched subpopulation with a frequency of approximately 50% within this subpopulation. Analysis of peripheral blood cells from WT-reconstituted mice stained for T cell, B cell, and myeloid markers confirmed that GFP positive cells were CD3+ or CD19+ (Mac-1-negative) lymphocytes. Overall these results demonstrate that GFPi is a bona fide RAG1/2 reporter substrate in mice and humans, *ex vivo* and *in vivo*.

### GFPi is a faithful classifier of RSS

**Figure 6:**
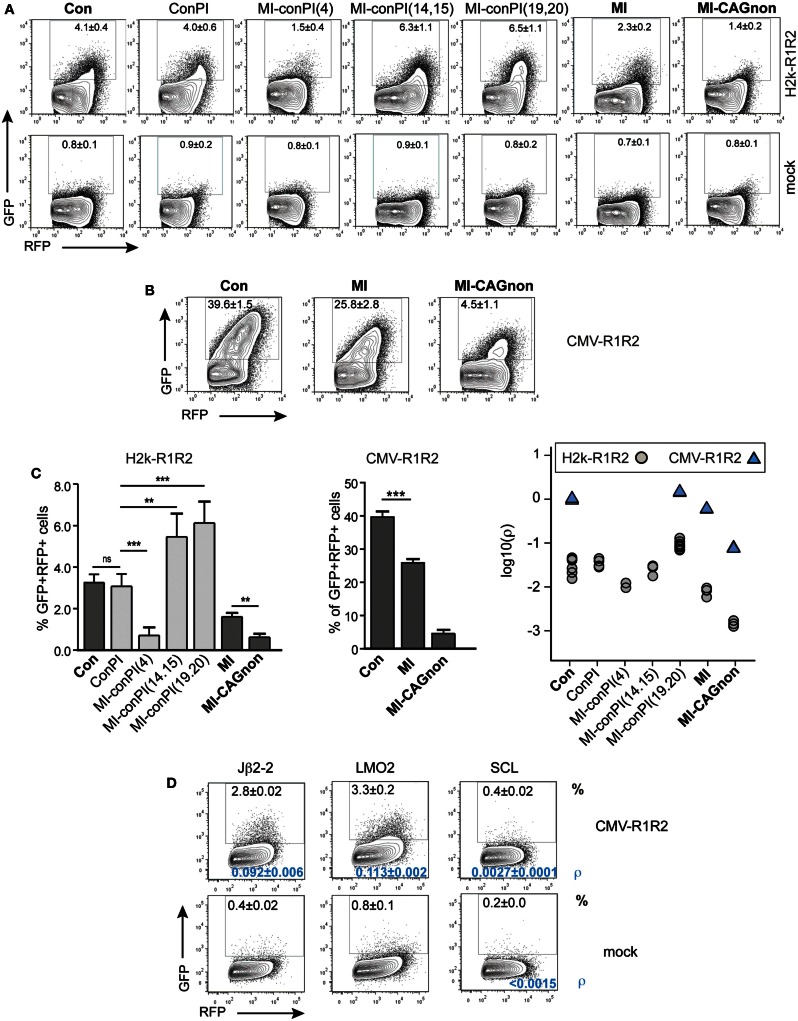
**The GFPi assay efficiently ranks nucleotide sequences for their RSS functionality**. GFPi variants carrying different 12 or 23-RSSs were tested in 293T cells transfected alone (mock) or co-transfected with either H2k-RAG1/2 **(A)** or CMV-RAG1/2 **(B,D)**. **(A–D)** GFPi 23-RSS variants Con23, ConPI, MI-conPI(4), MI-conPI(14,15), MI-conPI(19,20), MI, and MI-CAGnon were described previously (Cowell et al., 2004) and their nucleotide sequences are listed in Table 1. **(A,B)** Representative FACS analysis gated on RFP^+^ cells. Number are% of GFP^+^ cells **C**) Recombination efficiency defined as [(%GFP^+^ in test) – (%GFP^+^ in respective control)] (left and middle panel) At least six replicates from at least two independent experiments. Right panel shows the relative recombination coefficient ρ normalized to GFPi-Con. For MI-CAGnon, the less efficient RSS, ρ in the mock was <5.10^−5^ **D**) FACS analysis of 293T cells transfected with CMV-RAG1/2 and GFPi variants carrying 12-RSS of poor efficiencies paired with Con23. Jβ2-2 is found at the TCRβ locus, LMO2 and SCL are putative cRSS. Mock is Jβ2-2 transfected alone. The frequency of GFP^+^RFP^+^ cells in an RFP^+^ gate is indicated in the upper part of the plot and the recombination coefficient ρ relative to GFPi-Con in A, for which ρ = 1.0 ± 0.2) in the bottom part, in blue. Standard deviation of three replicates and their significance vs. the mocks. (**p < 0.05, ***p < 0.001, ns – not significant).

We next determined whether the GFPi reporter was able to classify RSSs according to their RE *in vitro*, similarly to the pJH290 system (Lewis et al., 1988). Six variants of the GFPi reporter were generated all containing the same 12-RSS Con12 paired with specific 23-RSS: ConPI, MI, MI-conPI(4), MI-conPI(14,15), MI-conPI(19,20), and MI-CAGnon, each differing in the 23-spacer and/or the nonamer sequence (Cowell et al., 2004), as detailed in Table 1. When cells were co-transfected with H2k-RAG1/2, the GFPi assay discriminated each 23-RSS along a range of GFP^+^RFP^+^ cell frequency from 0.5 to 6% (Figure 6A). Hence, the assay was sensitive to nucleotide changes in the spacer sequence and to the inhibitory effect of the specific CAGnon nonamer (MI vs. MI-CAGnon). Our ranking of these 23-RSS was overall consistent with previous results making use of the p290T reporter (Cowell et al., 2004), with the exception of the GFPi variants carrying the MI and MI-conPI(4) RSSs. As the 23-RSS containing the MI spacer was described as presenting the highest *in silico* score, according to the RSS Information Content (RIC score), and the highest experimental RE amongst all 23-RSSs tested (Cowell et al., 2004), we were particularly surprised by its low score in our assay. As the CMV-RAG GFPi system appeared to be ideal to study RSSs with low RE, we also tested MI, Con23, and MI-CAGnon in these conditions (Figure 6B) and confirmed that MI has low RE when compared to the classical consensus sequence. The ranking of this set of 23-RSS either by frequency of rearranged cells or by the coefficient of recombination ρ was reproducible across independent experiments (Figure 6C).

We next tested the capacity of the assay to detect 12-RSS of very low functionality. These were paired with 23-RSS Con23 and tested in conditions of high levels of RAG expression (Figure 6D). The frequency of GFP^+^RFP^+^ cells were of 2.3 ± 0.02% for the mouse TCR-Jβ2-2 12-RSS, a sequence previously reported to be a poor RSS when compared to a large set of V(D)J associated 12-RSS (Cowell et al., 2003). Putative cryptic 12-RSS (cRSS) have been described in the LMO2 and the SCL genes and proposed to be involved in oncogenic translocations or deletions found in some T-ALL. We could not detect rearrangement when scoring the SCL sequence by simple frequency of GFP^+^ cells. However the recombination coefficient ρ was low but higher than the respective background (SCL construct, no RAG). This result is in agreement with a previous study (Raghavan et al., 2001). The LMO2 sequence tested positive and scored similarly to the Jβ2-2 sequence. Together, these results confirm that the GFPi reporter assay is suitable to assess RSS and cRSS functions.

### GFPi adaptation to large-scale candidate RSS cloning

**Figure 7:**
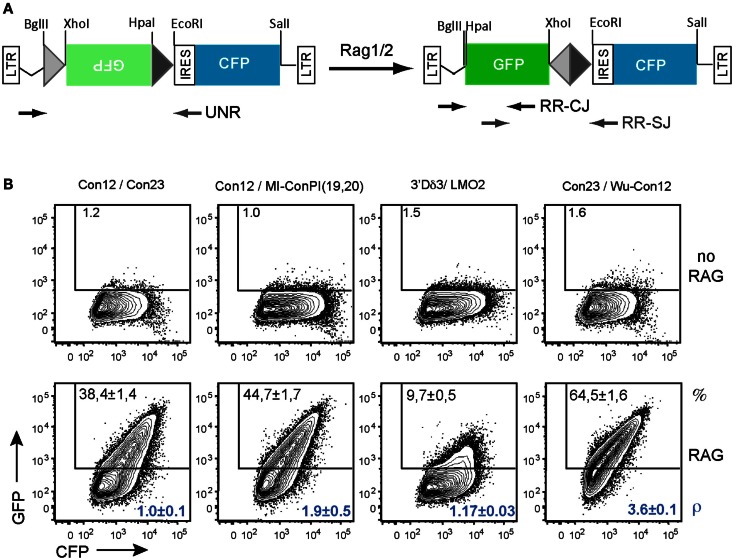
***In vitro* recombination assay using the GFPi-CFP variant**. **(A)** Linear representation of the GFPi-CFP reporter. **(B)** The recombination of different RSSs pairs was determined upon 293T cells transfection with each GFPi-CFP variants together with the CMV-RAG1/2 expressing plasmids. The 23-RSS Con23 and MI-conPI(19,20), as well as the 12-RSS Con12 and LMO2 were as in Figure 5. Wu-Con12 is a consensus 12-RSS defined by bio-informatics (Ramsden et al., 1994). The 3′Dδ3 is a physiological partner of LMO2 described in Dik et al. (2007). The specific pairs of RSS tested are indicated above the corresponding FACS plots and the sequences listed in Table 2. The recombination efficiency defined as (% GFP^+^ cells in test -% GFP^+^ cells in control) is indicated in the upper part of each plot. The relative recombination coefficient ρ, when con12/con23 is 1, is indicated in the bottom part of each plot, in blue. four replicates of two independent experiments.

The results above indicating that GFPi offers a fast and reliable readout of RAG activity prompted us to modify the original GFPi-mRFP reporter to ease the preparations of variants. We generated GFPi-CFP (Figure 7A), in which the indicator of transfection efficiency is provided by expression of CFP, offering an alternative color for eventual analysis in combination with other fluorescent reporters. To improve the efficiency of RSS insertions, GFPi-CFP was engineered to contain a pair of dissimilar cohesive-end cloning sites in 5′ and 3′ of GFP. Sequences under test were ordered as sense and antisense oligonucleotides bordered by the complementary cloning site ends. Annealing efficiency was optimal upon a temperature gradient thus allowing direct cloning into the digested and dephosphorylated GFPi-CFP. To further improve the GFPi efficiency each (putative) RSS sequences contained and additional GGG in 5′ of the heptamer (Yu and Lieber, 1999). Together, these modifications resulted in an insertion of nine nucleotides between the RSS in 5′ of the inverted GFP and the Kozak sequence (Table 2), dramatically reducing the possibility that nucleotide excision during the processing of the coding ends would affect the GFP sequence, as we had observed with GFPi-RFP (Table 3).

To validate this new tool, we tested the RE of constructs carrying various pairs of RSS when co-transfected with CMV-RAG (Figure 7B). We first compared the 23-RSS con23 and MI-conPI(19,20), each positioned in 3′ of the inverted GFP and paired with the 12-RSS con12. Similarly to the results obtained with GFPi-mRFP (Figure 6A), MI-conPI(19,20) performed better than Con23 (RE 47.7 ± 1.7 vs. RE 38.4 ± 1.4) in the GFPi-CFP assay. We next compared Con12, a sequence widely used as a consensus 12-RSS but actually a physiological RSS found at the Kappa locus, with Wu-Con12, a real consensus 12-RSS defined by analysis of a large number of physiological V(D)J associated RSS (Ramsden et al., 1994). Strikingly, when paired with Con23, Wu-Con12 performed much better than Con12 (% of GFP^+^ cells 65.5 ± 1.6 vs. 38.4 ± 1.4), confirming that it is a better-defined consensus 12-RSS. Finally, we tested the LMO2 12-cRSS suspected to mediate translocation to the TCR α/δ locus (Dik et al., 2007). This sequence, when paired with one of its natural partner in malignant translocations, the 23-RSS in 5′ of Dδ3, performed remarkably well, with a frequency of GFP^+^ cells of 9.7 ± 0.5, a result consistent with previous studies (Raghavan et al., 2001). With these analyses, we conclude that the GFPi assay, now also available as GFPi-CFP will be a suitable tool to test (putative) RSS properties on a medium to large scale.

## Discussion

Studies on V(D)J recombination and RAG-mediated genomic instability have relied on tools that provide information on the targeting of candidate substrates by RAG. RAG-mediated recombination reporters are included in those tools as they are key for further characterization of the regulation levels underlying the recruitment of a RSS or a cRSS. In this work, we introduce a reporter assay that combines a direct measurement of transfection/transduction efficiency and of RAG activity without the need for further procedures and we validated it as a tool for quantitative analyses.

Our fast method of readout, relying on a fluorescence-based reporter system to measure RAG activity is not novel. Recently, Scott et al. (2010) have generated the first mutually exclusive dual-fluorescence reporter that accounts a transfection efficiency marker as control but lacks the retroviral structure that enables the stable integration of the reporter. Accounting for differences in both methodologies, we observed that GFPi performs at one-log greater efficiency when tested with the same 12 and 23-RSS sequences. Another construct similar to GFPi, pMX-RSS-GFP-IRES-CD4, is a retroviral-based inversion reporter; that has been used *ex vivo* in primary mouse lymphoid cells, in studies of RAG mutant activities and regulation of recombination impacting on allelic exclusion (Liang et al., 2002; Lutz et al., 2011). When integrated, this reporter reaches recombination efficiencies similar to the ones of GFPi in the same context and with the same RSSs. Yet, contrarily to GFPi, this tool requires indirect detection of CD4 expression by antibody staining. Zheng and Schwarz (2006) have described the pE19HK-N, an exclusively episomal GFP-fluorescence inversion reporter in which GFP expression is under the control of the EF-1alpha promoter while the transfection efficiency is assessed by monitoring expression of an independently CMV-driven hemagglutinin gene, by antibody staining. Contrary to GFPi, transcription of each marker in pE19HK-N is controlled independently, which may lower the accuracy of the inferred recombination efficiencies. When transfecting 293 cells with RAG in this assay, recombination frequencies were of 0.6%. Other recombination reporters lack a second marker for transfection control. A mouse transgenic for the VEX-GFP recombination substrate was generated for *in vivo* detection of RAG activity (Borghesi et al., 2004). This system served as a RAG lineage tracer, as the RAG-dependent inversion of VEX would render cells VEX^+^. However, in this case, the system did not allow discriminating truly non-recombining cells from those carrying the silenced transgene. Arnal et al. have used several versions of RAG-mediated deletional reporters that conditioned the expression of GFP. These were used *in vitro* in the context of NHEJ and homologous recombination repair (Arnal et al., 2010). In both cases, the performance of these reporters cannot be directly compared to the one of GFPi. When analyzing all RAG-mediated reporter systems, none of the former combined all features presented by GFPi, namely: (1) a double-fluorescence system; (2) its usage in transient or stable assays as an episomal or retroviral substrate; (3) high resolution; (4) a tool here thoroughly validated for RAG and RSS studies.

We have shown here that GFPi can be used as a semi-quantitative assay by measuring the frequency of GFP+ cells as a proxy of the RE. This straightforward measurement can be obtained directly from the standard flow cytometry software. Nevertheless, the dynamic range of this measurement is limited, and as an alternative we proposed a rigorous quantitative method to estimate the relative recombination coefficient (i.e., the probability that a target episome is recombined) in the GFPi assay. The method provides estimates that span about four orders of magnitude and seem to be both accurate and precise. The variance within the coefficients estimates in independent transfection replicates is remarkably small when compared to the range of the estimates. It is noteworthy that this method is formally analogous to the classical limiting dilution analysis (LDA). The RFP signal and the apparent recombination coefficient (ρ_a_) in each cell is mathematically analogous to the cellular input and expected responder frequency in LDA (Carneiro et al., 2009). In this formal analogy each cell in the GFPi assay represents an independent replicate culture in the LDA, which allows to compute accurate and precise frequencies from very large number of “replicates” (>1000), which are obviously prohibitive when performing limiting dilutions. For this reason our method is effectively better with frequencies of negative cells that are in the range 30–100% in contrast with LDA, which operates better in the range 0–50%. This provides an intuition for why our method seems to be able to resolve recombination coefficients across several orders of magnitude with relatively high precision.

As a tool that scores RAG activity, GFPi will be useful for the study of mutant RAGs, and can serve as a functional diagnosis of Rag mutation in patients with primary immunodeficiencies, as previously done with other reporters (Schwarz et al., 1996; De Ravin et al., 2010). In agreement with this proposition, we demonstrated that GFPi reveals both mouse and human RAG activities. The former was evidenced in transfected fibroblasts, in *ex vivo* cultures of pre-B cells and during hematopoiesis *in vivo* while the latter was tested in human cell lines of lymphoid origin. As a tool to detect endogenous RAG activity *in vivo*, GFPi has the potential to serve in lineage-tracing and cell fate studies in hematopoiesis. The high resolution and double reporting features of GFPi also make it suitable to address *in vivo* the lingering questions of physiological RAG expression and activity in non-lymphoid cells (Chun et al., 1991) and to further elucidate the conditions that may promote RAG re-expression in mature lymphocytes (Hikida et al., 1996) and in lymphoid or non-lymphoid tumors (Gashaw et al., 2005; Marculescu et al., 2006; Schlissel et al., 2006; McIntyre et al., 2007).

We have validated GFPi as a tool able to reveal RSSs and cRSSs, scoring each according to their RE *in vitro*. We have used GFPi to score a set of synthetic 23-RSSs and detected the effect of single nucleotide changes in the spacer sequence as well as action of the inhibitory non-amer. Our ranking for this set of sequences was similar to that established previously, in a study relying on an assay that differs considerably from ours (Cowell et al., 2002). While the former work quantified rearranged molecules using the classical bacterial selection assay, we measured cells undergoing rearrangement. Moreover, we used a fibroblast cell line transiently expressing controlled levels of exogenous RAG to run our GFPi assay while they transfected the 103/BCL2 mouse pre-B cell that express endogenous RAG at more heterogeneous levels. We document that the GFPi assay is able to detect rare events of recombination involving poor 12-RSS and cRSS substrates. Faithfull scoring of non-RSS sequences is more challenging but may be required, for instance when designing gene therapy vectors. In the course of this work, we tested another putative cRSS sequence (not shown) that scored negative *in vitro*. This negative result was confirmed by absence of amplicons when performing controlled nested PCR around the coding and the SJ on plasmid DNA extracted from these assays. We take this example as an indication that the GFPi assay is also reliable in identifying non-RSS sequences and has therefore the required resolution for testing candidate cRSS substrates potentially implicated in genomic instability.

In conclusion, the GFPi system is here validated to pave the way for future studies. It will be a convenient tool to reassess the nucleotide definition of a RAG target sequence. In the present work we have isolated the relative contribution of RAG expression levels and the RSS nucleotide sequence to GFPi RE. The GFPi *in vitro* assay should provide a way to disentangle other components responsible for the overall efficiency of the reaction. For instance systematic scoring of defined RSS with the GFPi and comparison with their frequency of recruitment in physiological conditions should shed light on the role of specific epigenetic marks in the modulation of RAG-mediated recombination. Along another line of thoughts, the GFPi assay may also be useful in evolutionary studies of RAGs and RSSs. Finally, the retroviral version of GFPi could be most valuable for assessing real-time events of recombination either *ex vivo* by classical imaging techniques or *in vivo* by intravital imaging approaches.

## Conflict of Interest Statement

The authors declare that the research was conducted in the absence of any commercial or financial relationships that could be construed as a potential conflict of interest.

## References

[B1] Arnal S. M. Holub A. J. Salus S. S. Roth D. B. (2010). Non-consensus heptamer sequences destabilize the RAG post-cleavage complex, making ends available to alternative DNA repair pathways. Nucleic Acids Res. 38, 2944–2954 10.1093/nar/gkp1252 20139091 PMC2875030

[B2] Barreto V. Marques R. Demengeot J. (2001). Early death and severe lymphopenia caused by ubiquitous expression of the Rag1 and Rag2 genes in mice. Eur. J. Immunol. 31, 3763–3772 10.1002/1521-4141(200112)31:12<3763::AID-IMMU3763>3.0.CO;2-Y 11745397

[B3] Bassing C. H. Swat W. Alt F. W. (2002). The mechanism and regulation of chromosomal V(D)J recombination. Cell 109, S45–S55 10.1016/S0092-8674(02)00675-X 11983152

[B4] Borghesi L. Hsu L. Y. Miller J. P. Anderson M. Herzenberg L. Schlissel M. S. (2004). B lineage-specific regulation of V(D)J recombinase activity is established in common lymphoid progenitors. J. Exp. Med. 199, 491–502 10.1084/jem.20031802 14769852 PMC2211824

[B5] Campbell R. E. Tour O. Palmer A. E. Steinbach P. A. Baird G. S. Zacharias D. A. (2002). A monomeric red fluorescent protein. Proc. Natl. Acad. Sci. U.S.A. 99, 7877–7882 10.1073/pnas.062425699 12060735 PMC122988

[B6] Carneiro J. Duarte L. Padovan E. (2009). Limiting dilution analysis of antigen-specific T cells. Methods Mol. Biol. 514, 95–105 10.1007/978-1-60327-527-9_7 19048215

[B7] Carvalho T. L. Mota-Santos T. Cumano A. Demengeot J. Vieira P. (2001). Arrested B lymphopoiesis and persistence of activated B cells in adult interleukin 7(-/-) mice. J. Exp. Med. 194, 1141–1150 10.1084/jem.194.8.1141 11602642 PMC2193519

[B8] Chun J. J. Schatz D. G. Oettinger M. A. Jaenisch R. Baltimore D. (1991). The recombination activating gene-1 (RAG-1) transcript is present in the murine central nervous system. Cell 64, 189–200 10.1016/0092-8674(91)90220-S 1986864

[B9] Cowell L. G. Davila M. Kepler T. B. Kelsoe G. (2002). Identification and utilization of arbitrary correlations in models of recombination signal sequences. Genome Biol. 3:research0072 10.1186/gb-2002-3-12-research0072 12537561 PMC151174

[B10] Cowell L. G. Davila M. Ramsden D. Kelsoe G. (2004). Computational tools for understanding sequence variability in recombination signals. Immunol. Rev. 200, 57–69 10.1111/j.0105-2896.2004.00171.x 15242396

[B11] Cowell L. G. Davila M. Yang K. Kepler T. B. Kelsoe G. (2003). Prospective estimation of recombination signal efficiency and identification of functional cryptic signals in the genome by statistical modeling. J. Exp. Med. 197, 207–220 10.1084/jem.20020250 12538660 PMC2193808

[B12] De Ravin S. S. Cowen E. W. Zarember K. A. Whiting-Theobald N. L. Kuhns D. B. Sandler N. G. (2010). Hypomorphic Rag mutations can cause destructive midline granulomatous disease. Blood 116, 1263–1271 10.1182/blood-2009-07-233734 20489056 PMC2938237

[B13] Dik W. A. Nadel B. Przybylski G. K. Asnafi V. Grabarczyk P. Navarro J. M. (2007). Different chromosomal breakpoints impact the level of LMO2 expression in T-ALL. Blood 110, 388–392 10.1182/blood-2006-12-064816 17360939

[B14] Gashaw I. Grummer R. Klein-Hitpass L. Dushaj O. Bergmann M. Brehm R. (2005). Gene signatures of testicular seminoma with emphasis on expression of ets variant gene 4. Cell. Mol. Life Sci. 62, 2359–2368 10.1007/s00018-005-5250-9 16158187 PMC11139191

[B15] Gauss G. H. Domain I. Hsieh C. L. Lieber M. R. (1998). V(D)J recombination activity in human hematopoietic cells: correlation with developmental stage and genome stability. Eur. J. Immunol. 28, 351–358 10.1002/(SICI)1521-4141(199801)28:01<351::AID-IMMU351>3.0.CO;2-# 9485214

[B16] Gellert M. (2002). V(D)J recombination: RAG proteins, repair factors, and regulation. Annu. Rev. Biochem. 71, 101–132 10.1146/annurev.biochem.71.090501.150203 12045092

[B17] Hesse J. E. Lieber M. R. Gellert M. Mizuuchi K. (1987). Extrachromosomal DNA substrates in pre-B cells undergo inversion or deletion at immunoglobulin V-(D)-J joining signals. Cell 49, 775–783 10.1016/0092-8674(87)90615-5 3495343

[B18] Hikida M. Mori M. Takai T. Tomochika K. Hamatani K. Ohmori H. (1996). Reexpression of RAG-1 and RAG-2 genes in activated mature mouse B cells. Science 274, 2092–2094 10.1126/science.274.5295.2092 8953042

[B19] Komori T. Okada A. Stewart V. Alt F. W. (1993). Lack of N regions in antigen receptor variable region genes of TdT-deficient lymphocytes. Science 261, 1171–1175 10.1126/science.8356451 8356451

[B20] Lewis S. M. Hesse J. E. Mizuuchi K. Gellert M. (1988). Novel strand exchanges in V(D)J recombination. Cell 55, 1099–1107 10.1016/0092-8674(88)90254-1 3144437

[B21] Liang H. E. Hsu L. Y. Cado D. Cowell L. G. Kelsoe G. Schlissel M. S. (2002). The “dispensable” portion of RAG2 Is necessary for efficient V-to-DJ rearrangement during B and T cell development. Immunity 17, 639–651 10.1016/S1074-7613(02)00448-X 12433370

[B22] Lutz J. Heideman M. R. Roth E. van den Berk P. Muller W. Raman C. (2011). Pro-B cells sense productive immunoglobulin heavy chain rearrangement irrespective of polypeptide production. Proc. Natl. Acad. Sci. U.S.A. 108, 10644–10649 10.1073/pnas.1019224108 21670279 PMC3127927

[B23] Marculescu R. Vanura K. Montpellier B. Roulland S. Le T. Navarro J. M. (2006). Recombinase, chromosomal translocations and lymphoid neoplasia: targeting mistakes and repair failures. DNA Repair (Amst.) 5, 1246–1258 10.1016/j.dnarep.2006.05.015 16798110

[B24] McIntyre A. Summersgill B. Lu Y. J. Missiaglia E. Kitazawa S. Oosterhuis J. W. (2007). Genomic copy number and expression patterns in testicular germ cell tumours. Br. J. Cancer 97, 1707–1712 10.1038/sj.bjc.6604079 18059402 PMC2360290

[B25] Oettinger M. A. Schatz D. G. Gorka C. Baltimore D. (1990). RAG-1 and RAG-2, adjacent genes that synergistically activate V(D)J recombination. Science 248, 1517–1523 10.1126/science.2360047 2360047

[B26] Oltz E. M. Alt F. W. Lin W. C. Chen J. Taccioli G. Desiderio S. (1993). A V(D)J recombinase-inducible B-cell line: role of transcriptional enhancer elements in directing V(D)J recombination. Mol. Cell. Biol. 13, 6223–6230 8413222 10.1128/mcb.13.10.6223-6230.1993 PMC364681

[B27] Pear W. S. Aster J. C. Scott M. L. Hasserjian R. P. Soffer B. Sklar J. (1996). Exclusive development of T cell neoplasms in mice transplanted with bone marrow expressing activated Notch alleles. J. Exp. Med. 183, 2283–2291 10.1084/jem.183.5.2283 8642337 PMC2192581

[B28] Raghavan S. C. Kirsch I. R. Lieber M. R. (2001). Analysis of the V(D)J recombination efficiency at lymphoid chromosomal translocation breakpoints. J. Biol. Chem. 276, 29126–29133 10.1074/jbc.M103797200 11390401

[B29] Ramsden D. A. Baetz K. Wu G. E. (1994). Conservation of sequence in recombination signal sequence spacers. Nucleic Acids Res. 22, 1785–1796 10.1093/nar/22.10.1785 8208601 PMC308075

[B30] Rizzo M. A. Springer G. H. Granada B. Piston D. W. (2004). An improved cyan fluorescent protein variant useful for FRET. Nat. Biotechnol. 22, 445–449 10.1038/nbt945 14990965

[B31] Roose J. P. Diehn M. Tomlinson M. G. Lin J. Alizadeh A. A. Botstein D. (2003). T cell receptor-independent basal signaling via Erk and Abl kinases suppresses RAG gene expression. PLoS Biol. 1:E53 10.1371/journal.pbio.0000053 14624253 PMC261890

[B32] Sarmento L. M. Huang H. Limon A. Gordon W. Fernandes J. Tavares M. J. (2005). Notch1 modulates timing of G1-S progression by inducing SKP2 transcription and p27 Kip1 degradation. J. Exp. Med. 202, 157–168 10.1084/jem.20050559 15998794 PMC2212905

[B33] Schatz D. G. Oettinger M. A. Baltimore D. (1989). The V(D)J recombination activating gene, RAG-1. Cell 59, 1035–1048 10.1016/0092-8674(89)90760-5 2598259

[B34] Schlissel M. S. Kaffer C. R. Curry J. D. (2006). Leukemia and lymphoma: a cost of doing business for adaptive immunity. Genes Dev. 20, 1539–1544 10.1101/gad.1446506 16778072

[B35] Schwarz K. Gauss G. H. Ludwig L. Pannicke U. Li Z. Lindner D. (1996). RAG mutations in human B cell-negative SCID. Science 274, 97–99 10.1126/science.274.5284.97 8810255

[B36] Scott G. B. de Wynter E. A. Cook G. P. (2010). Detecting variable (V), diversity (D) and joining (J) gene segment recombination using a two-colour fluorescence system. Mob. DNA 1, 9 10.1186/1759-8753-1-9 20226006 PMC3225881

[B37] Tonegawa S. (1983). Somatic generation of antibody diversity. Nature 302, 575–581 10.1038/302575a0 6300689

[B38] Yu K. Lieber M. R. (1999). Mechanistic basis for coding end sequence effects in the initiation of V(D)J recombination. Mol. Cell. Biol. 19, 8094–8102 10567535 10.1128/mcb.19.12.8094 PMC84894

[B39] Zheng X. Schwarz K. (2006). Making V(D)J rearrangement visible: quantification of recombination efficiency in real time at the single cell level. J. Immunol. Methods 315, 133–143 10.1016/j.jim.2006.07.012 16935293

